# *IL-10* diplotype and septic shock: Genotype–Phenotype associations with hemodynamic dysfunction and IL-10 expression

**DOI:** 10.3389/fmolb.2026.1958117

**Published:** 2026-10-14

**Authors:** Amr A. Amin, Mohammad Althubiti, Sameer H. Fatani, Ahmad H. Mufti, Zohor A. Azher, Ghaidaa M. Malibari, Neda M. Bogari

**Affiliations:** 1 Department of Biochemistry, Faculty of Medicine, Umm Al-Qura University, Makkah, Saudi Arabia; 2 Faculty of Medicine, Ain-Shams University, Cairo, Egypt; 3 Department of Medical Genetics, Faculty of Medicine, Umm Al-Qura University, Makkah, Saudi Arabia

**Keywords:** haplotype, hemodynamic dysfunction, IL-10 (Interleukin 10), interleukin, sepsis, septic shock

## Abstract

**Introduction:**

Early identification of patients with sepsis who are at increased risk of developing septic shock remains a major clinical challenge. Although genetic variation within the *IL-10* locus has been associated with immune regulation, the relationship between the rs1518111–rs1554286 haplotype architecture, inflammatory phenotype, and hemodynamic status has not been fully characterized. This study investigated whether *IL-10* haplotypes are associated with progression to septic shock and their relationship with blood pressure and systemic and urinary IL-10 concentrations.

**Methods:**

In this prospective observational study, 492 adult patients with sepsis admitted to the intensive care unit were enrolled according to the Sepsis-3 criteria. Genotyping of *IL-10* rs1518111 and rs1554286 was performed using TaqMan allelic discrimination assays. Linkage disequilibrium, haplotypes, and diplotypes were reconstructed using Haploview and PHASE software. Serum and urinary IL-10 concentrations were quantified by enzyme-linked immunosorbent assay. Associations between genetic variants, clinical phenotypes, and septic shock were evaluated using multivariable logistic regression adjusted for age, sex, admission white blood cell count, and Sequential Organ Failure Assessment score. Diagnostic performance was assessed using receiver operating characteristic (ROC) analysis.

**Results:**

The two loci demonstrated strong linkage disequilibrium (*D′* = 1.00, *r* ^*2*^ = 0.903). Compared with the reference heterozygous diplotype (C-G/T-A), the homozygous variant diplotype (T-A/T-A) was independently associated with septic shock (adjusted OR, 6.30; 95% CI, 3.68–10.78; *P* < 0.001), whereas the homozygous wild-type diplotype (C-G/C-G) was associated with lower odds (adjusted OR, 0.28; 95% CI, 0.14–0.61; *P* < 0.001). Variant homozygotes exhibited higher serum and urinary IL-10 concentrations and lower blood pressure than wild-type carriers. Serum IL-10 yielded an AUC of 0.851, while a combined clinical-genetic model achieved an AUC of 0.889 (95% CI, 0.820–0.958).

**Conclusion:**

Variation within the IL-10 rs1518111–rs1554286 Diplotype block was associated with susceptibility to septic shock, higher IL-10 concentrations, and lower arterial blood pressure in patients with sepsis. Integration of *IL-10* diplotype information with routinely available clinical and laboratory variables modestly improved discrimination for septic shock. These findings support further evaluation of *IL-10* genetic variation as a complementary biomarker for risk stratification in larger, multicenter cohorts.

## 1 Introduction

Sepsis is defined under the Sepsis-3 consensus as life-threatening organ dysfunction arising from a dysregulated host response to infection (Singer et al., 2016), with its most severe phenotype, septic shock, representing refractory hemodynamic failure and carrying a high (30%–50%) ICU mortality rate (La et al., 2025; Pettilä et al., 2026; Zhu and He, 2026). Early prediction of circulatory collapse remains a critical gap (La et al., 2025; Rudd et al., 2020) driven by a breakdown in the fine equilibrium between pro-inflammatory amplification and immunoregulatory counter-suppression (Delano and Ward, 2016; Ma et al., 2025), a process mediated centrally by Interleukin-10 (IL-10) (Hotchkiss et al., 2016; Fiorentino et al., 1991; Moore et al., 2001). Although IL-10 is essential for limiting collateral tissue injury, its excessive or prolonged expression can contribute to systemic immunoparalysis, potentially increasing susceptibility to secondary infections and mortality (Wang et al., 2026; Xing et al., 2023); notably, *IL-10* expression can also be influenced by genetic variation (Turner et al., 1997; Crawley et al., 1999). The *IL-10* gene (chromosome 1q31-q32) harbors substantial variation, where classical promoter single-nucleotide polymorphisms (SNPs)—such as −1082A/G, −819T/C, and −592A/C—alter transcription regulation and disease susceptibility (Crawley et al., 1999; Hofmann et al., 2012; Hayat et al., 2024; Sun et al., 2022), but focus has recently shifted to less-characterized intronic regulatory variants (rs1518111 and rs1554286) (Qaddourah et al., 2014; Amin et al., 2024). Because single-locus evaluations underestimate genetic risk, modeling adjacent variants in linkage disequilibrium (LD) as functional haplotypes and diploid combinations (diplotypes) provide superior statistical power and biological resolution (Bonnen et al., 2002). Crucially, Middle Eastern populations remain underrepresented in genomic reference resources, limiting the interpretation of population-specific variation (Ghorbani et al., 2025) and have failed to pair multi-site genomic models with parallel systemic (serum) and compartmentalized (urinary) IL-10 expressions to capture localized inflammatory dynamics. To our knowledge, few studies have evaluated the combined haplotype and diplotype architecture of rs1518111 and rs1554286 in relation to septic shock and paired systemic and urinary IL-10 phenotypes. This study aimed to evaluate this combined haplotype and diplotype of rs1518111 and rs1554286, correlating these genomic configurations directly with septic shock risk and quantitative systemic and renal cytokine phenotypes in a critically ill cohort.

## 2 Materials and methods

### 2.1 Study design and setting

This prospective observational genetic association study was conducted in the intensive care units (ICUs) of a tertiary care medical center. Consecutive adult patients with sepsis were recruited between August 2023 and September 2025. The study cohort comprised a newly recruited, independent population with no participant overlap with our previously published investigations of *IL-10* polymorphisms in sepsis-associated acute kidney injury, which were based on a separate historical cohort recruited between May 2016 and October 2018 (Amin et al., 2024). The present study was specifically designed to investigate the association of IL-10 rs1518111–rs1554286 haplotypes and diplotypes with progression to septic shock. Study design, conduct, and reporting followed the STREGA (STrengthening the REporting of Genetic Association Studies) recommendations, an extension of the STROBE statement for genetic association studies (Little et al., 2009). The study was prospectively registered, and all procedures were performed in accordance with the Declaration of Helsinki.

### 2.2 Participants

During the prospective study period, consecutive adults (≥18 years) admitted to the ICU with suspected or documented infection were screened for eligibility. Patients were included if they met the Third International Consensus Definitions for Sepsis and Septic Shock (Sepsis-3) (Singer et al., 2016). Sepsis was defined as suspected or documented infection accompanied by an acute increase of ≥2 points in the Sequential Organ Failure Assessment (SOFA) score, indicating organ dysfunction. Septic shock was defined as sepsis with persistent hypotension requiring vasopressor therapy to maintain a mean arterial pressure (MAP) ≥65 mmHg and a serum lactate concentration >2 mmol/L despite adequate fluid resuscitation. Patient enrollment, clinical classification, and baseline blood and urine sampling were completed within 24 h of fulfilling these diagnostic criteria.

To eliminate systematic phenotypic bias and confounding biochemical signals driving sepsis progression, patients meeting any of the following pre-specified criteria were excluded:

1. Vulnerable Populations and Altered Physiology: Patients aged <18 years or those with confirmed/suspected pregnancy, due to distinct hemodynamic baselines and altered gestational immune kinetics.
2. Advanced Malignancy and Pre-Arrest Directives: Individuals with end-stage neoplastic disease (life expectancy <3 months) or active Do-Not-Resuscitate (DNR) orders, minimizing confounding from early non-septic mortality or the withdrawal of life-sustaining treatment.
3. Pre-Existing Immunosuppression: Baseline immune alterations capable of distorting cytokine kinetics, defined as: active *HIV* with *CD4* ^*+*^ T-lymphocyte count <200 cells/L; active post-transplantation immunosuppressive regimens; or chronic systemic corticosteroids (≥20 mg prednisone daily equivalent for >14 consecutive days).
4. End-Stage Renal Disease (ESRD): Patients on maintenance hemodialysis or peritoneal dialysis prior to admission, as chronic uremic states and regular renal replacement therapy alter baseline systemic and urinary cytokine clearance.
5. Prolonged Prior ICU Exposure: Transfers from external facilities with a total intensive care stay >72 h prior to screening, mitigating survival and lead-time bias.

### 2.3 Clinical endpoints and phenotypic data collection

The primary study endpoint was progression to septic shock during the index ICU admission, defined according to the Sepsis-3 criteria. Patients who fulfilled these criteria comprised the septic shock group, whereas patients with sepsis who did not require vasopressor support served as the non-shock reference group. Admission serum lactate was measured in all participants to confirm septic shock classification.

Baseline demographic, clinical, and laboratory data were collected from the electronic medical records using a standardized case report form. Variables included age, sex, white blood cell (WBC) count, systolic and diastolic blood pressure, Sequential Organ Failure Assessment (SOFA) score, serum creatinine, and blood urea nitrogen. Acute kidney injury (AKI) at admission was classified according to the Kidney Disease: Improving Global Outcomes (KDIGO) criteria using admission serum creatinine and clinical records. Serial lactate measurements were not available and were therefore not included in subsequent analyses.

### 2.4 Biological sample collection

Peripheral venous blood was collected into EDTA-anticoagulated vacutainer tubes within 24 h of sepsis confirmation. Simultaneously, a compartmentalized (24-h urine) sample was obtained. For cytokine analysis and DNA extraction from whole blood, blood was centrifuged at 3,000 × g for 10 min at 4 °C; serum was aliquoted and stored at −80 °C until batch analysis. Urine samples were centrifuged at 2,000 × g for 15 min to remove cellular debris, and clarified supernatants were stored at −80 °C.

### 2.5 Genomic DNA extraction and genotyping

Genomic DNA was isolated from whole blood using the QIAamp DNA Blood Mini Kit (Qiagen, Hilden, Germany). Yield and purity were verified via NanoDrop 2000 spectrophotometry (Thermo Fisher Scientific, Waltham, MA, USA), accepting an A260/A280 ratio of 1.8–2.0.

Genotyping of *IL-10* rs1518111 and rs1554286 was performed using fluorogenic 5′→3′exonuclease TaqMan SNP Genotyping Assays (Holland et al., 1991) (Applied Biosystems, Foster City, CA, USA) on a real-time PCR platform. Each 10 µl reaction contained 10 ng of template DNA, 2× TaqMan Genotyping Master Mix, and 40× FAM/VIC-labeled allele-specific primer-probe mix. Real-time PCR cycling comprised 95 °C for 10min, followed by 40 cycles of 95 °C for 15s and 60 °C for 60s (combined annealing and extension).

### 2.6 Quality control and genotype calling

To ensure genotyping integrity, laboratory personnel were blinded to clinical outcomes, each reaction plate incorporated negative no-template controls, and a random 15% subset of samples underwent independent replicate amplification with 100% concordance. Automated genotype assignment was executed using allelic discrimination software based on endpoint fluorescence clustering; ambiguous or low-signal samples were excluded. The overall genotyping call rate was calculated across the entire dataset, with a pre-specified plate-level repeat threshold of <95%. Genotyping reproducibility was additionally verified by re-running a random 10% subset of samples on independent plates. Hardy–Weinberg equilibrium (HWE) testing was conducted in the non-shock sepsis reference cohort to assess population genotype distributions, recognizing that disease-associated cohorts frequently exhibit HWE deviation due to risk-allele enrichment.

### 2.7 Cytokine quantification

Quantification Systemic (serum) and compartmentalized (24-h urine) IL-10 concentrations were quantified in duplicate via commercial sandwich ELISA (Quantikine Human IL-10; R&D Systems, Minneapolis, MN, USA). The assay detection limit was 3.9 pg/mL (range: 7.8–500 pg/mL), with intra- and inter-assay coefficients of variation strictly <5% and <10%, respectively. Serum samples were diluted 10-fold prior to measurement, and all final concentrations are expressed in pg/mL to permit direct comparative profiling. Crucially, absolute urinary *IL-10* levels were utilized without normalization to urinary creatinine. While creatinine standardization is conventional in stable ambulatory cohorts, this practice is fundamentally confounded in acute critical care settings characterized by rapid, dynamic shifts in glomerular filtration and tubular handling (Waiker et al., 2010).

### 2.8 Haplotype inference and diplotype assignment

Pairwise linkage disequilibrium (LD) between rs1518111 and rs1554286 was characterised using Haploview version 4.2 (Broad Institute, Cambridge, MA, USA) (Barrett et al., 2005), with computation of the standardised disequilibrium coefficient (*D′*), the squared allelic correlation coefficient (r^2^), and the logarithm-of-odds (LOD) score. Complete LD was defined by *D′* = 1.00.

Multi-locus haplotypes spanning both SNPs were inferred using the Expectation-Maximisation (EM) algorithm implemented in the PHASE v2.1 software (Stephens et al., 2001). Haplotype phase was assigned to each participant at a posterior probability threshold of >0.95. Individual diplotype configurations were then resolved from the phased output. Haplotypes and diplotypes with an estimated frequency below 1% in the overall cohort were excluded from association modelling to avoid sparse-cell instability.

**Missing data:** Missing data Missing observations across baseline clinical covariates and genotyping variables were infrequent (<5%). Given the low proportion of missing data, complete-case analysis was used for all inferential analyses. Under these circumstances, complete-case analysis is considered an appropriate approach and is unlikely to introduce meaningful bias when data are missing completely at random (MCAR). No imputation procedures were performed.

### 2.9 Statistical analysis

Statistical analyses were performed using IBM SPSS Statistics version 28.0 (IBM Corp., Armonk, NY, USA) and R software version 4.3.1 (R Foundation for Statistical Computing, Vienna, Austria). Continuous variables were evaluated for normality using the Shapiro–Wilk test and are presented as mean ± standard deviation (SD) or median (interquartile range [IQR]), with comparisons executed via Student’s t-test/one-way ANOVA or Mann–Whitney U/Kruskal–Wallis tests, respectively. Categorical variables are expressed as frequencies (percentages) and compared using Pearson’s chi2 or Fisher’s exact tests (<5 expected cell counts).

To prevent disease-associated genotype enrichment from introducing stratification bias, Hardy–Weinberg Equilibrium (HWE) compliance was tested strictly within the reference non-shock sepsis cohort via χ^2^ goodness-of-fit. Pairwise linkage disequilibrium (LD; Lewontin’s *D', r2*, and LOD) between *IL-10* rs1518111 and rs1554286 was estimated using Haploview version 4.2. Haplotype phase reconstruction and individual diplotype assignments were performed using the expectation-maximization algorithm in PHASE version 2.1, designating the heterozygous C-G/T-A diplotype as the baseline reference.

The independent association between genetic architectures and septic shock was modeled using multivariable logistic regression adjusted for admission covariates: age, sex, WBCs, and Sequential Organ Failure Assessment (SOFA) score. Multicollinearity and calibration were verified using Variance Inflation Factors (VIF <5) and the Hosmer–Lemeshow test, respectively. Discriminatory accuracy was quantified using Receiver Operating Characteristic (ROC) curves to compute Areas Under the Curve (AUC) with 95% confidence intervals (CIs).

All statistical tests were two-sided, with *P* < 0.05 considered significant. To account for multiple comparisons in genetic association testing, *P*-values were adjusted using the Benjamini–Hochberg procedure was used as a sensitivity/multiple-testing assessment.

## 3 Results

### 3.1 Study population characteristics

The prospective cohort comprised 492 adults with confirmed Sepsis-3, including 84 patients (17.1%) who progressed to septic shock and 408 (82.9%) who remained in the non-shock sepsis group. Baseline characteristics are summarized in Table 1. Sex distribution was comparable between the two groups, whereas age differed significantly, with patients who developed septic shock were younger than those with non-shock sepsis (56 [52–63] vs. 65 [55–74] years, respectively; *P* = 0.018). Patients with septic shock also exhibited greater clinical severity, including lower systolic blood pressure (106 ± 15 vs. 111 ± 12 mmHg; *P* = 0.004) and higher leukocyte counts (*P* < 0.001).

**TABLE 1:** Baseline demographic, clinical, and biochemical parameters of the study cohort.

Parameter	Non-shock sepsis (n = 408)	Septic shock (n = 84)	*P*-value
Demographics			
Age (years)	65 (55–74)	56 (52–63)	0.018*
Sex (Male), n (%)	240 (58.8%)	56 (66.7%)	0.181
Clinical Metrics			
WBC count (×10^9^/L)	14.3 (10.9–17.2)	17.4 (13.5–22.0)	<0.001*
Systolic BP (mmHg)	111 ± 12	106 ± 15	0.004*
Diastolic BP (mmHg)	71 ± 8	60 ± 11	<0.001*
Biomarkers			
Serum IL-10 (pg/mL) median (IQR)	169.4 (135.6–208.9)	193.9 (175.8–228.2)	<0.001*
Urinary IL-10 (pg/mL) median (IQR)	17.2 (14.8–19.1)	20.8 (16.5–24.2)	<0.001*

Normally distributed variables (blood pressures) are presented as Mean ± SD, and evaluated via Student's t-test. Non-normally distributed variables (Age, WBC, IL-10) are presented as Median (IQR) and compared using the Mann–Whitney U test. Categorical variables are evaluated via Pearson’s chi-square test. * Indicates significance.

Hemodynamic measurements were obtained using continuous invasive arterial monitoring in 68 of 84 patients with septic shock (81.0%), whereas automated oscillometric monitoring was used in all 408 patients with non-shock sepsis (100%). Consistent with the greater clinical severity of the shock group, both serum and urinary IL-10 concentrations were significantly higher in patients with septic shock than in those with non-shock sepsis (both *P* < 0.001).

### 3.2 Genotype and linkage disequilibrium structure

Within the overall cohort (n = 492), the minor allele (T) frequency of IL-10 rs1518111 was 0.402. Genotypes comprised 216 (43.9%) C/C, 156 (31.7%) C/T, and 120 (24.4%) T/T individuals. For rs1554286, the minor allele (A) frequency was 0.378, with 228 (46.3%) G/G, 156 (31.7%) G/A, and 108 (22.0%) A/A individuals (Table 2).

**TABLE 2:** Genotypic distributions, Allele Frequencies, and HWE validation.

Polymorphism	Total cohort (N = 492)	Non-shock sepsis (n = 408)	Septic shock (n = 84)	Non-shock sepsis HWE (*P*)
rs1518111				
C/C, n (%)	216 (43.9%)	204 (50.0%)	12 (14.3%)	<0.001^*^
C/T, n (%)	156 (31.7%)	144 (35.3%)	12 (14.3%)	
T/T, n (%)	120 (24.4%)	60 (14.7%)	60 (71.4%)	
Minor allele (T) Freq	0.402	0.323	0.785	
rs1554286				
G/G, n (%)	228 (46.3%)	216 (52.9%)	12 (14.3%)	0.00245^*^
G/A, n (%)	156 (31.7%)	144 (35.3%)	12 (14.3%)	
A/A, n (%)	108 (22.0%)	48 (11.8%)	60 (71.4%)	
Minor allele (A) Freq	0.378	0.294	0.785	

In the non-shock sepsis group, both rs1518111 and rs1554286 showed significant deviation from Hardy–Weinberg equilibrium (both *P* < 0.001 and *P* = 0.00245). Because the reference group was not a population-based control sample, these deviations were interpreted cautiously and were not taken as evidence of genotyping validity in isolation. Genotyping quality was instead supported by blinded laboratory procedures, negative controls, replicate testing, and high reproducibility. Pairwise analysis demonstrated strong linkage disequilibrium between rs1518111 and rs1554286, with D′ = 1.00 and r^2^ = 0.903. The strong linkage supported subsequent reconstruction of phased haplotypes and diplotypes for joint association analysis (Table 3).

**TABLE 3:** Reconstructed Multi-locus Diplotype configurations and Septic Shock risk.

Diplotype architecture	Non-shock sepsis (n = 408)	Septic shock (n = 84)	aOR (95% CI)	*P*-value
C-G/T-A (heterozygous)	120 (29.4%)	24 (28.6%)	1.00	—
C-G/C-G (protective)	192 (47.1%)	11 (13.09%)	0.28 (0.14–0.61)	<0.001
T-A/T-A (high risk)	48 (11.8%)	37 (44.0%)	6.3 (3.68–10.78)	<0.001
Discordant/Rare profiles	48 (11.8%)	12 (14.3%)	1.25 (0.55–2.84)	0.59

Adjusted Odds Ratios (aOR) were estimated using multivariate logistic regression with the C-G/T-A diplotype as the reference category, adjusting for prespecified covariates; age, sex, admission WBC, and baseline SOFA, score. Frequencies are shown for descriptive purposes only; adjusted effect estimates are not expected to equal crude odds ratios calculated from the contingency table.

### 3.3 Single-variant and multi-locus diplotype associations with septic shock

Multivariable logistic regression under a recessive model was used to evaluate the associations of homozygous variant carriage with septic shock after adjustment for age, sex, admission white blood cell (WBC) count, and baseline SOFA score. Homozygous rs1518111 T/T carriage was associated with increased odds of septic shock (adjusted OR [aOR] = 3.63; 95% CI, 1.81–7.28), as was rs1554286 A/A carriage (aOR = 1.96; 95% CI, 1.00–3.84) (Figure 1). Given the strong linkage disequilibrium between the two loci, phased haplotypes and corresponding diplotypes were subsequently evaluated (Table 3). The homozygous T-A/T-A diplotype was independently associated with septic shock relative to the C-G/T-A reference configuration (aOR = 6.30; 95% CI, 3.68–10.78; *P* < 0.001). Conversely, the C-G/C-G diplotype was associated with lower odds of septic shock (aOR = 0.28; 95% CI, 0.14–0.61; *P* < 0.001). The heterozygous C-G/T-A configuration, present in 29.4% of the cohort, was not associated with increased risk relative to the reference configuration (aOR = 0.96; 95% CI, 0.42–2.18; *P* = 0.939). Thus, the observed association was concentrated among individuals homozygous for the T-A haplotype rather than being attributable simply to carriage of a T-A haplotype.

**FIGURE 1:**
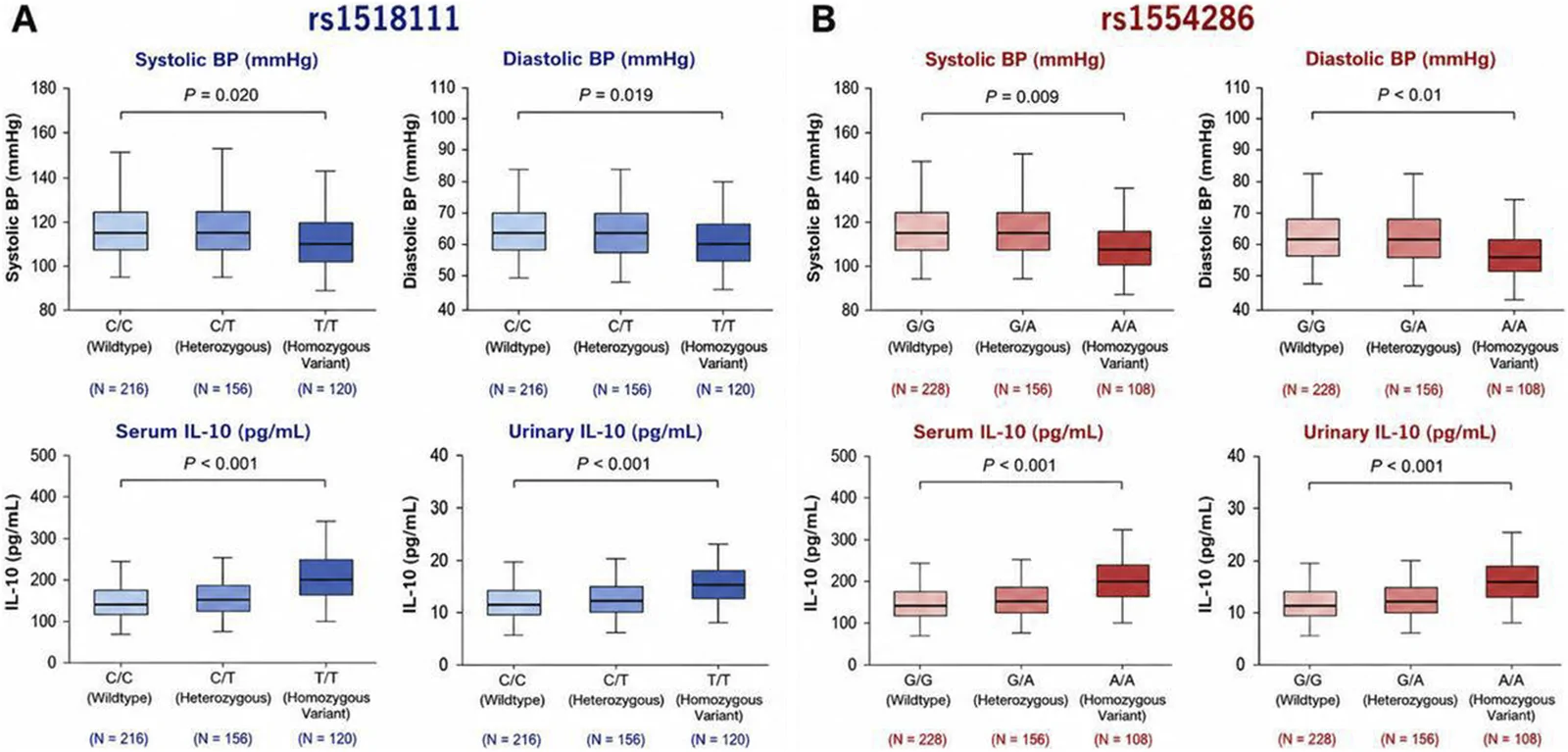
Distribution of systolic blood pressure (SBP), diastolic blood pressure (DBP), serum IL-10, and urinary IL-10 concentrations according to **(A)** rs1518111 and **(B)** rs1554286 genotype groups. Upper panels show baseline SBP and DBP, whereas lower panels present serum and urinary IL-10 concentrations. Homozygous variant carriers (rs1518111 T/T and rs1554286 A/A) exhibited lower blood pressure measurements and higher serum and urinary IL-10 concentrations than wild-type carriers. Overall comparisons among genotype groups were assessed using one-way ANOVA for SBP and DBP and the Kruskal–Wallis test for serum and urinary IL-10 concentrations. Pairwise comparisons for blood pressure are indicated above the corresponding panels.

### 3.4 Genotype–*IL-10* expression associations

The relationship between *IL-10* genotype and IL-10 concentrations was evaluated across both serum and urinary compartments (Table 4; Figure 2). For both rs1518111 and rs1554286, IL-10 concentrations differed significantly across genotype groups, with homozygous minor-allele carriers (T/T and A/A, respectively) exhibiting the highest serum and urinary IL-10 concentrations (Kruskal–Wallis global *P* < 0.001 for both loci).

**TABLE 4:** Genotype-Phenotype Mapping: Hemodynamic and IL-10 levels stratified by genetic architecture.

Genetic locus	N (492)	Systolic BP (mmHg) Mean ± SD	Diastolic BP (mmHg) Mean ± SD	Serum IL-10 (pg/mL) Median (IQR)	Urinary IL-10 (pg/mL) Median (IQR)
rs1518111					
C/C (Wildtype)	216	119.9 ± 12.8	75.3 ± 8.6	165.8 (132.5–212.6)	16.6 (14.9–19.3)
C/T (heterozygous)	156	119.5 ± 13.1	75.0 ± 8.8	171.2 (139.5–208.7)	17.1 (14.3–19.0)
T/T (homozygous variant)	120	111.5 ± 16.5	69.5 ± 11.5	214.6 (173.9–292.5)	19.5 (16.9–24.1)
*Overall P*-value		*P* = 0.020	*P* = 0.019	<0.001	<0.001
rs1554286					
G/G (Wildtype)	228	120.0 ± 12.8	75.3 ± 8.6	165.2 (129.1–208.9)	16.6 (14.8–19.2)
G/A (heterozygous)	156	119.5 ± 13.1	75.0 ± 8.8	171.4 (139.3–208.2)	17.1 (14.3–19.0)
A/A (homozygous variant)	108	110.4 ± 16.6	68.8 ± 11.6	214.3 (175.7–312.7)	19.6 (17.1–24.4)
*P*-value		*P* = 0.009	*P* < 0.01	<0.001	<0.001

Normally distributed hemodynamic variables (systolic and diastolic blood pressure) are presented as mean ± standard deviation (SD) and were compared across genotype groups using one-way analysis of variance (ANOVA). Serum and urinary IL-10, concentrations exhibited non-normal distributions and are therefore presented as median (interquartile range, IQR); comparisons among genotype groups were performed using the Kruskal–Wallis test. Reported P-values represent overall differences across the three genotype categories. Where appropriate, *post hoc*pairwise comparisons were multiplicity-adjusted using Tukey’s test (ANOVA) or Dunn’s test (Kruskal–Wallis) with adjustment for multiple comparisons.

**FIGURE 2:**
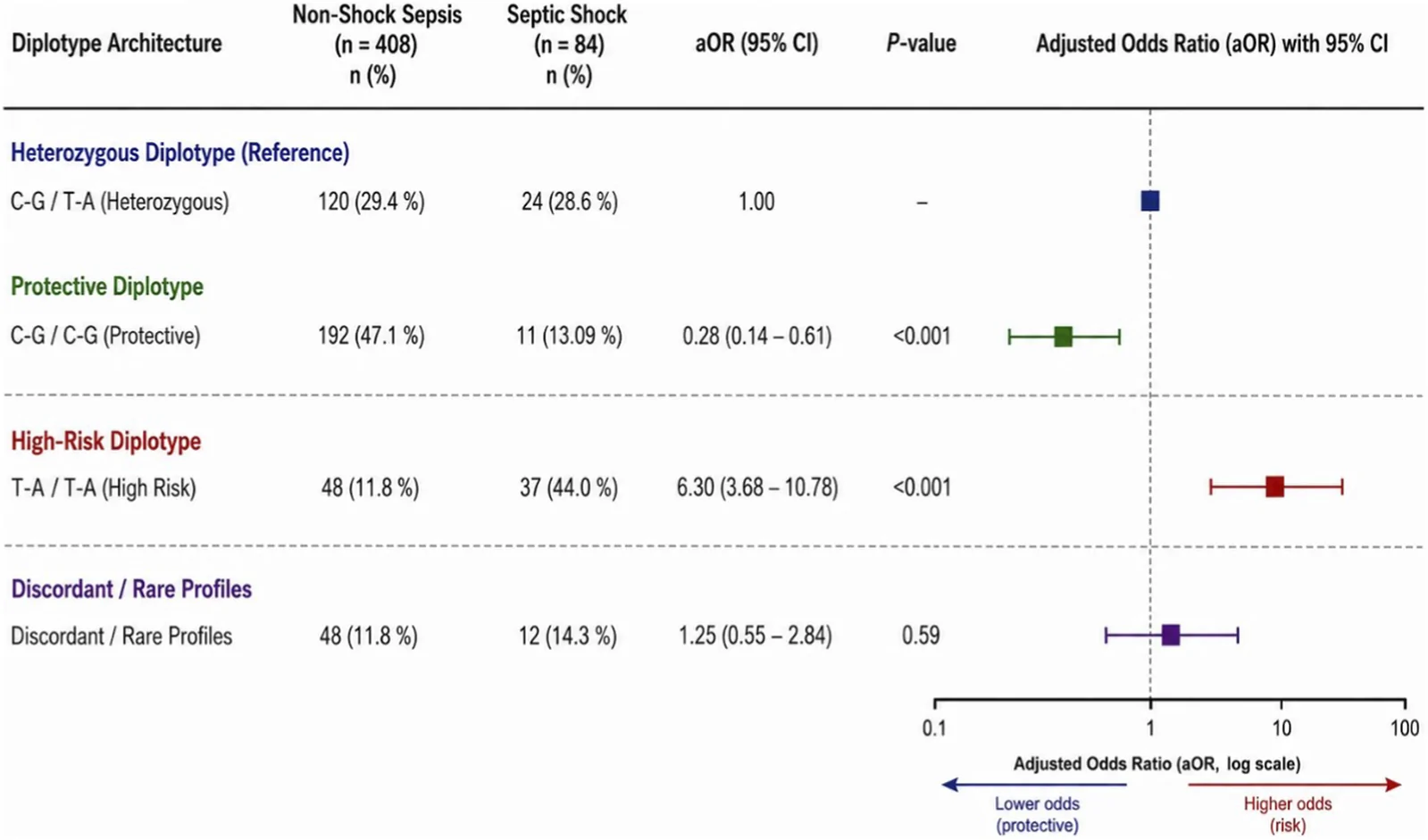
Forest plot illustrates adjusted odds ratios (aORs) and 95% confidence intervals (CIs) for the association between reconstructed *IL-10* diplotype configurations and progression to septic shock. Odds ratios were estimated using multivariable logistic regression with adjustment for age, sex, admission white blood cell count, and baseline Sequential Organ Failure Assessment (SOFA) score. The heterozygous C-G/T-A diplotype served as the reference category (aOR = 1.00). Horizontal lines represent 95% confidence intervals and squares represent adjusted point estimates plotted on a logarithmic scale. An adjusted odds ratio <1 indicates lower odds of septic shock relative to the reference group, whereas values > 1 indicate higher odds.

Genotype-associated differences were also observed for hemodynamic measures. Across both loci, systolic and diastolic blood pressures differed significantly among genotype groups (ANOVA global *P* < 0.001). Post-hoc comparisons showed lower arterial pressures among homozygous variant carriers compared with wild-type individuals for rs1518111 (SBP, *P* = 0.020; DBP, *P* = 0.019) and rs1554286 (SBP, *P* = 0.009; DBP, *P* = 0.009), whereas heterozygous genotypes did not differ significantly from wild-type genotypes.

Correlation analyses further showed concordant variation between WBC counts, IL-10 concentrations, and septic shock status. Serum and urinary IL-10 concentrations also showed parallel variation. In contrast, age and renal indices, including serum urea and creatinine, demonstrated weaker relationships with the principal disease-severity measures. Collectively, these analyses demonstrate an observed association among *IL-10* genotype, IL-10 concentrations, leukocyte count, and hemodynamic status, without establishing a causal relationship among these variables.

### 3.5 Multivariable associations and predictive performance

Multivariable logistic regression was performed to determine whether IL-10 concentrations and genetic variables remained associated with septic shock after adjustment for chronological age, sex, admission WBC count, and baseline SOFA score (Table 5; Figure 3). Serum IL-10 remained independently associated with septic shock, with an adjusted OR of 1.22 per IQR increase (95% CI, 1.11–1.33; *P* = 1.36 × 10^−5^). Homozygous rs1518111 T/T carriage (aOR = 3.63), rs1554286 A/A carriage (aOR = 1.96), and the homozygous T-A/T-A diplotype (aOR = 6.30) also remained associated with septic shock in the multivariable models.

**TABLE 5:** ROC Discriminative accuracy for predicting septic shock.

Diagnostic model parameter	AUC	95% CI	Sensitivity (%)	Specificity (%)
Combined clinical-genomic model	0.889	0.820–0.958	95.2	72.5
Serum IL-10 alone (>210 pg/mL)	0.851	0.808–0.894	95.2	69.6
Urinary IL-10 alone	0.736	0.679–0.793	71.4	68.6
White blood cell count	0.744	0.686–0.802	71.4	70.6

AUC: area under the curve; CI: Confidence Interval. the combined model integrates diplotype risk mapping, WBC, counts, and systemic IL-10, concentrations. Optimal diagnostic thresholds were established by maximizing Youden’s index.

**FIGURE 3:**
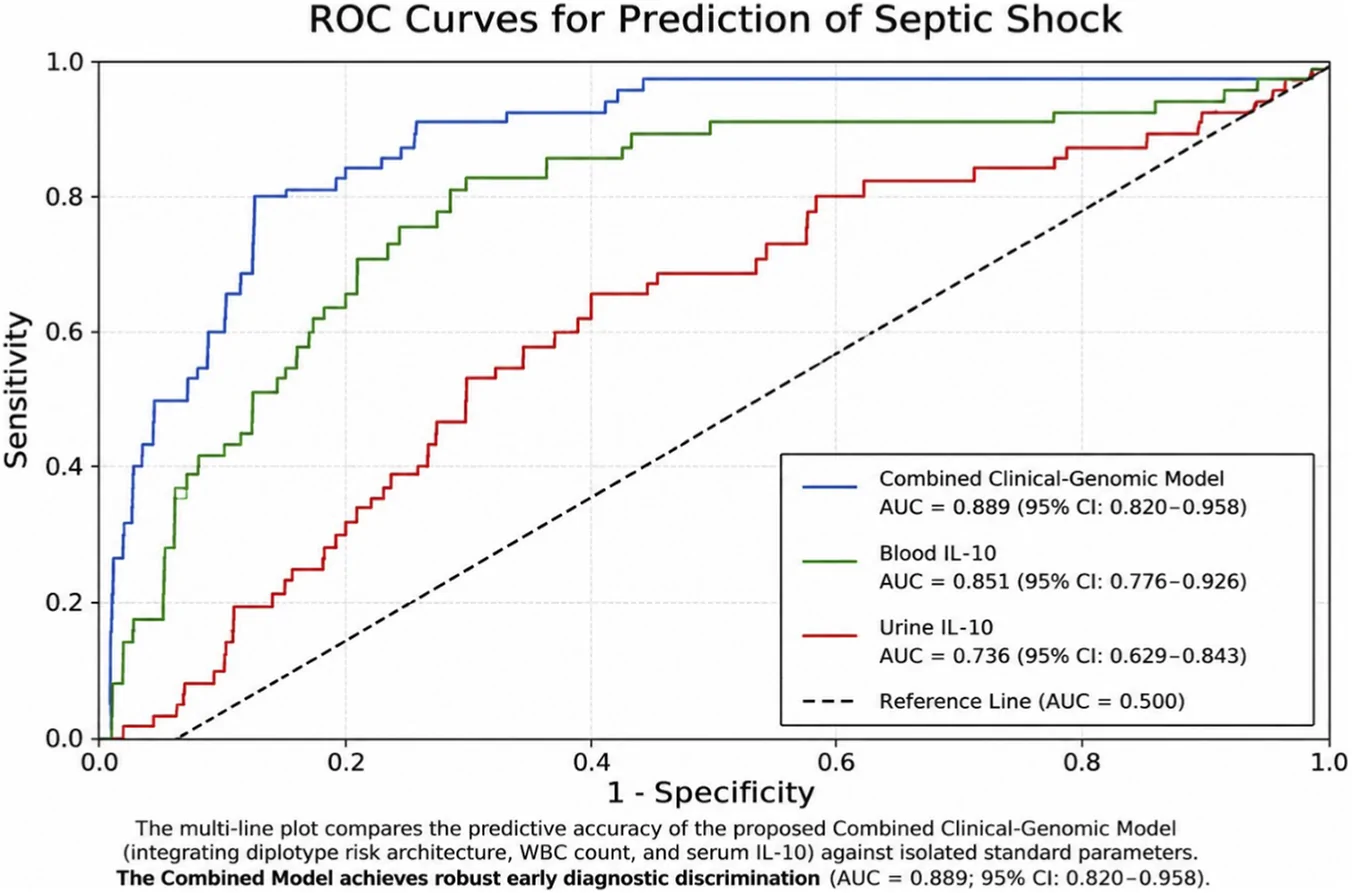
The multi-line plot compares the predictive accuracy of the individual biomarker (IL-10 expression) versus the Combined Model (IL-10 expression + *IL-10* diplotype + SOFA score) across ascending deciles of septic shock risk. The Combined Model achieves early diagnostic discrimination, significantly outperforming IL-10 alone in correctly identifying high-risk patient subgroups.

Receiver operating characteristic (ROC) analysis was used to evaluate the discriminatory performance of individual biomarkers and the combined model. Serum *IL-10* showed the highest AUC among the individual variables evaluated (AUC = 0.851). At a threshold of 210 pg/mL, serum *IL-10* yielded 95.2% sensitivity, 69.6% specificity, and a negative predictive value of 98.6%. The corresponding AUCs were 0.736 for urinary *IL-10*, 0.744 for WBC count.

A combined model incorporating serum *IL-10*, admission WBC count, and diplotype categories showed higher apparent discriminatory performance (AUC = 0.889; 95% CI, 0.820–0.958), with 95.2% sensitivity and 72.5% specificity. Because these performance estimates were derived within the study cohort without bootstrap correction, cross-validation, or external validation, they should be considered preliminary and require confirmation in an independent population.

## 4 Discussion

This prospective genetic association study identifies regulatory variation across the *IL-10* rs1518111–rs1554286 locus as a potential contributor to clinical hemodynamic instability and compartmentalized cytokine expression during progression from sepsis to septic shock. The findings indicate that: (i) homozygous variant genotypes are significantly overrepresented among patients progressing to septic shock; (ii) the co-inherited homozygous T-A/T-A diplotype is associated with septic shock after adjustment for admission severity; (iii) risk-allele carriage is associated with higher serum and urinary IL-10 concentrations together with lower systemic blood pressure; and (iv) integration of genetic diplotypes with baseline clinical parameters provides good discrimination for septic shock (AUC = 0.889; 95% CI, 0.820–0.958). Although age differed significantly between the septic shock and non-shock groups, sex distributions were comparable; age was therefore considered as a baseline covariate in multivariable analyses where applicable. Collectively, these findings support a potential role for inherited variation within this *IL-10* linkage block in modulating anti-inflammatory responses and hemodynamic dysfunction during sepsis progression.

### 4.1 Genetic association of IL-10 variants with septic shock

The present study identified substantially different distributions of *IL-10* rs1518111 and rs1554286 genotypes between patients with non-shock sepsis and those who progressed to septic shock. Homozygous carriage of rs1518111 T/T and rs1554286 A/A was markedly enriched among patients with septic shock, and both associations remained significant after adjustment for age, sex, admission WBC count, and baseline SOFA score. These findings are broadly consistent with previous evidence that *IL-10* genetic variation may contribute to heterogeneity in severe infection, but the genetic architecture observed here differs from that examined in much of the earlier literature. Previous investigations have focused predominantly on classical promoter polymorphisms, particularly −1082 A/G, −819 T/C, and −592 A/C, with inconsistent associations across populations and clinical settings (Gao et al., 2015; Gong et al., 2014; Schaaf et al., 2003). A 2023 meta-analysis likewise demonstrated substantial heterogeneity in the relationship between *IL-10* polymorphisms and sepsis susceptibility, indicating that individual variants do not show uniform effects across different study populations (Zhang et al., 2023). The present findings therefore extend, rather than simply reproduce, the existing literature by examining rs1518111 and rs1554286 in relation specifically to progression from established sepsis to septic shock.

Several factors may explain the difference between the present results and earlier reports. First, the phenotype examined here was septic shock rather than sepsis susceptibility, thereby focusing on the transition from established infection-associated organ dysfunction to circulatory failure. Second, the variants investigated in the present study are distinct from the classical promoter polymorphisms most frequently evaluated previously, and their effects may depend on the local linkage disequilibrium structure and the genetic background of the study population. Third, differences in ancestry, sepsis definitions, illness severity, clinical management, and sampling time can materially affect the apparent magnitude of genetic associations. This is particularly relevant because sepsis encompasses biologically heterogeneous host-response states, ranging from predominantly inflammatory phenotypes to states characterized by marked immune suppression or immunoparalysis (Zhu and He, 2026). Recent work on sepsis biomarkers and precision medicine similarly emphasizes that biological heterogeneity and biomarker kinetics can limit the reproducibility and clinical interpretation of individual biomarkers when measured without consideration of disease stage (Wang et al., 2025).

The magnitude of the associations observed in the present cohort should therefore be interpreted in the context of the specific phenotype and population studied. The accompanying genotype-stratified differences in serum and urinary IL-10 concentrations provide biological context for the genetic associations, but they do not establish that either variant directly determines cytokine production or circulatory failure. Instead, the concordance between genotype and phenotype supports further investigation of these variants as potential markers of inter-individual variation in the host response to severe sepsis.

The recurrence of rs1518111 in our previous study of sepsis-associated acute kidney injury (S-AKI) provides an additional point of comparison (Amin et al., 2024). That study, conducted in an independent cohort, also identified rs1518111 variation together with serum and urinary IL-10 concentrations as associated with S-AKI. The replication of rs1518111 association across two clinically distinct sepsis-related phenotypes is noteworthy, although the outcomes are not interchangeable. In the earlier study, the endpoint was renal injury, whereas the present analysis focused on progression to septic shock. The recurrence may therefore indicate that rs1518111 warrants investigation across different severe-sepsis phenotypes, but it cannot establish a common causal pathway. Importantly, the independent cohorts and absence of participant overlap strengthen the rationale for replication while still requiring confirmation in larger, prospectively recruited populations.

Overall, the present findings are most appropriately interpreted as evidence for a context-dependent association between *IL-10* genetic variation and severe sepsis phenotype. The differences from earlier studies highlight the importance of variant selection, ancestry, phenotype definition, and disease stage when evaluating cytokine polymorphisms. The results consequently support further validation of rs1518111 and rs1554286 rather than suggesting that these variants represent universal genetic determinants of septic shock.

### 4.2 Linkage disequilibrium and diplotype architecture

The strong linkage disequilibrium between rs1518111 and rs1554286 (*D′* = 1.00; *r* ^*2*^ = 0.903) provides an important context for interpreting their individual associations. The present study identified a predominant set of linked configurations together with a smaller group of discordant or rare profiles comprising 60 individuals (12.2%; Table 3). The combination of D′ and r^2^ indicates strong non-random association between the two loci at the population level, whereas the observed discordant profiles demonstrate that they cannot be regarded as completely interchangeable at the individual level. This distinction is important when comparing the present diplotype findings with single-SNP analyses, because the apparent effect of either variant may partly reflect its correlation with the other locus.

Within this genetic structure, the homozygous T-A/T-A diplotype was independently associated with septic shock compared with the C-G/T-A reference configuration (aOR = 6.30; 95% CI, 3.68–10.78; *P* < 0.001), whereas C-G/C-G was associated with lower odds of septic shock (aOR = 0.28; 95% CI, 0.14–0.61; *P* < 0.001). In contrast, the C-G/T-A configuration showed no evidence of increased risk (aOR = 0.96; 95% CI, 0.42–2.18; *P* = 0.939). The discordant/rare profiles were therefore retained as a distinct component of the observed genetic structure and were not assumed to share the risk characteristics of the major diplotype categories in the absence of direct statistical evidence, consistent with the need to account for multilocus haplotype structure when evaluating locus-level associations (Stephens et al., 2001).

The diplotype findings provide an additional perspective beyond the individual SNP associations. The stronger association observed for T-A/T-A compared with either single-locus estimate suggests that the joint configuration may capture genetic information not fully represented by modeling the two variants independently. However, the present data cannot determine whether this represents a true biological interaction between the loci, an effect of another functional variant in linkage with this region, or the statistical consequence of the underlying haplotype structure. This distinction is particularly important because strong LD can cause correlated variants to show similar associations without demonstrating that either variant is itself the functional allele.

The absence of an association among C-G/T-A carriers should likewise not be interpreted as definitive evidence of a biological recessive mechanism. The observed pattern may be compatible with a stronger association among individuals carrying two T-A haplotypes, but the modest cohort size and presence of discordant/rare profiles limit inference regarding the underlying inheritance model. Larger cohorts with direct haplotype phasing, broader regional sequencing, and independent replication will be required to determine whether the observed T-A/T-A association represents a reproducible diplotype-specific effect or reflects the population distribution of linked variants.

The broader genetic context is also relevant to interpretation. Contemporary approaches to precision medicine in sepsis increasingly recognize that clinical phenotypes may reflect combinations of genetic background, immune-state biomarkers, and disease-stage characteristics rather than single molecular determinants (Wang et al., 2025). In this context, the present diplotype analysis should be viewed as a hypothesis-generating attempt to capture multilocus genetic structure associated with septic shock rather than as evidence of a defined functional haplotype mechanism.

### 4.3 Genotype–phenotype correlation: IL-10 expression and blood pressure

An important feature of the present study is the concordance between *IL-10* genotype, circulating IL-10 concentrations, and hemodynamic phenotype. Homozygous rs1518111 T/T and rs1554286 A/A carriers exhibited the highest serum and urinary IL-10 concentrations and lower systolic and diastolic blood pressures than the corresponding non-homozygous groups (Table 4; Figure 2). This pattern provides phenotypic support for the genetic associations observed in the case-control analysis, although the cross-sectional measurement of IL-10 prevents determination of whether increased IL-10 preceded, accompanied, or resulted from the development of septic shock.

The serum findings are broadly compatible with previous work showing that circulating IL-10 concentrations can discriminate clinically important sepsis-related phenotypes. In an independent cohort of critically ill patients with sepsis-associated acute kidney injury, Amin et al. reported associations of both serum and urinary IL-10 concentrations with S-AKI and found that the rs1518111 TT genotype was independently associated with the outcome (Amin et al., 2024). The concordance between that study and the present findings is notable because both investigated the same genetic locus and measured IL-10 in both serum and urine, despite examining different clinical outcomes. This supports the possibility that rs1518111-associated variation in IL-10 levels may be relevant across more than one severe-sepsis phenotype. However, the studies cannot establish that the same biological pathway accounts for AKI and septic shock, and differences in outcome definition, patient characteristics, and disease severity may contribute to the observed associations.

The present serum IL-10 findings should also be considered against the broader biomarker literature. Reviews of genetic polymorphisms and biomarkers in septic shock emphasize that IL-10 and other inflammatory mediators reflect disease severity and immune-state heterogeneity, but their diagnostic and prognostic performance varies according to clinical context and timing of measurement (Wu et al., 2024). The present study measured IL-10 during an early period after sepsis confirmation and therefore captures a relatively narrow phase of the host response. Studies using later sampling points, serial measurements, or different sepsis phenotypes may consequently report different effect sizes or discriminatory performance. The lack of serial measurements in the present study prevents direct assessment of whether genotype-associated IL-10 differences persist, increase, or resolve during clinical progression.

The relationship between IL-10 and hypotension is biologically plausible but should likewise be interpreted cautiously. IL-10 is an important counter-regulatory cytokine involved in modulation of inflammatory signaling, immune-cell activity, and prevention of tissue injury (Moore et al., 2001; Hofmann et al., 2012). Increased IL-10 concentrations during severe sepsis may therefore accompany a shift toward an immunoregulatory state. However, the present observational data cannot establish that *IL-10* excess directly caused the lower blood pressure observed among carriers of the variant homozygous genotypes. Hemodynamic deterioration in septic shock is multifactorial and is influenced by vasodilation, endothelial dysfunction, myocardial function, intravascular volume, infection characteristics, and treatment. Accordingly, the genotype–*IL-10*–blood pressure relationship observed here should be regarded as a coordinated clinical phenotype rather than proof of a single mechanistic pathway.

The urinary findings provide an additional, potentially useful dimension. Serum and urinary IL-10 concentrations showed parallel variation in the present cohort, and the previous S-AKI study similarly reported substantial discriminatory performance for both compartments (Amin et al., 2024). Nevertheless, urinary cytokine concentrations are influenced by renal function, filtration, tubular handling, and local renal inflammatory processes. The current findings therefore support urinary IL-10 as a candidate compartment for further investigation rather than establishing it as a validated surrogate of systemic *IL-10* activity. This distinction is particularly important in sepsis, where renal dysfunction is common and may independently alter urinary biomarker behavior.

Taken together, the present results agree with previous evidence that IL-10 concentrations and *IL-10* genetic variation may provide information about heterogeneity among patients with severe sepsis, while also demonstrating that the magnitude and clinical expression of these associations depend on the phenotype being examined. The agreement with the independent S-AKI cohort strengthens the rationale for further investigation of rs1518111, whereas the differences between studies emphasize the need to account for disease stage, clinical endpoint, population background, and biomarker kinetics. Current evidence therefore supports the *IL-10* genotype–cytokine relationship as a promising research signal, but not yet as a validated mechanism or clinical biomarker.

### 4.4 Strengths, limitations, and clinical implications

The major strength of this investigation lies in its integrated clinical-genomic phenotyping, combining multi-locus diplotype analysis with paired systemic and urinary IL-10 measurements and hemodynamic characterization. The study also incorporated stringent laboratory quality-control procedures, including blinded laboratory processing, negative controls, replicate amplification, and reproducibility testing, providing additional support for the reliability and consistency of the genotyping results.

Several limitations should be acknowledged. **First**, IL-10 concentrations were measured at a single early time point, within 24 h of sepsis confirmation. Although this sampling window provides an assessment of the early host-response phenotype, it does not permit evaluation of longitudinal cytokine trajectories or establish the temporal relationship between IL-10 concentrations and subsequent septic shock. Accordingly, the observed associations should not be interpreted as evidence of causality. **Second**, this was a single-center study conducted in a specific patient population, which may limit the generalizability of the observed allele, genotype, and haplotype frequencies and their associations with septic shock to other ethnic, geographic, and clinical populations. Population-specific linkage disequilibrium patterns, clinical characteristics, sepsis case mix, and differences in patient management may affect the magnitude and reproducibility of these associations. The study was also not designed to determine whether these genetic associations are specific to septic shock or extend to other severe sepsis-related outcomes. Independent replication in larger, multicenter cohorts will therefore be necessary to assess their consistency and external validity. **Third**, the biomarker assessment was focused on *IL*-*10* and did not include several established markers of inflammation, immune dysfunction, and tissue hypoperfusion, including *IL*-*6, TNF-α*, procalcitonin, monocyte *HLA-DR*, and serial lactate clearance. Serial lactate measurements were not available in the study dataset. This study was deliberately focused on the *IL-10* genetic–cytokine–hemodynamic axis, whereas broader inflammatory, immune, and metabolic profiling represents an important direction for future investigation. **Fourth**, the combined clinical-genetic model was derived and evaluated within the same cohort and was not externally validated. The observed AUC of 0.889 therefore reflects preliminary discrimination within this study population and should not be interpreted as evidence of clinical readiness. External validation in larger, prospectively recruited cohorts is required before the model can be considered for clinical application.

From a clinical perspective, the observed associations between *IL-10* diplotype architecture, IL-10 concentrations, and hemodynamic phenotype suggest that these genetic markers may warrant evaluation as potential adjuncts to established clinical and biomarker-based approaches to risk stratification. However, the present study does not establish that *IL*-*10* genotype can predict circulatory collapse before its clinical manifestation, nor does it provide evidence that genotype-informed management improves outcomes. Although contemporary sepsis guidance emphasizes early recognition and management of hemodynamic instability (Ma et al., 2025), the clinical utility of incorporating *IL*-*10* diplotypes into such assessment remains to be determined. Prospective studies should therefore evaluate whether adding genetic information to clinical and biochemical predictors improves discrimination, calibration, and clinical decision-making beyond existing models.

At present, *IL-10* genotyping should not be used to guide vasopressor selection or other bedside treatment decisions. Larger multi-center and multi-ethnic cohorts with serial biomarker measurements, independent model validation, and functional investigation are needed to determine the reproducibility and biological significance of the observed associations. Until such evidence is available, the findings should be regarded as hypothesis-generating and supportive of further investigation rather than as a basis for changes in clinical practice.

The baseline clinical and biochemical comparison of the study cohorts (Table 1) reveals a distinct physiological partition between patients who remain hemodynamically stable and those who transition to refractory circulatory failure. Demographic parameters such as age demonstrate a highly significant difference (*P* = 0.018), with younger patients disproportionately progressing to shock. Patients who develop septic shock present with a significantly more advanced inflammatory and hemodynamic state at ICU, admission. This is demonstrated by marked systemic hypotension, with a mean systolic blood pressure of 106 ± 15 mmHg compared to 111 ± 12 mmHg in the non-shock group, and a mean diastolic blood pressure of 60 ± 11 mmHg compared to 71 ± 8 mmHg (*P* < 0.005 and *P* < 0.001, respectively). This circulatory compromise is accompanied by a significant difference in baseline white blood cell counts, which are elevated in the shock cohort (median 17.4 × 109/L vs. 14.3 × 109/L; *P* < 0.001).

Molecularly, progression to septic shock is characterized by highly significant, parallel elevations in both circulating and localized cytokine concentrations. Median serum IL-10, levels are substantially higher in septic shock patients than in those with non-shock sepsis (193.9 pg/mL vs. 169.4 pg/mL; *P* < 0.001). This systemic increase is mirrored in the renal compartment, where Median urinary IL-10, levels were also significantly higher in the septic shock group than in the non-shock group (20.8 vs. 17.2 pg/mL; *P* < 0.001). The parallel elevation of this immunoregulatory cytokine in both serum and urine suggests that the counter-regulatory immune response is activated across different physiological compartments, pointing to a coordinated, genetically determined driver of cytokine overproduction.

Table 2 summarizes the genotype distributions, allele frequencies, and HWE, assessment for rs1518111 and rs1554286. Both variants showed marked enrichment of the minor alleles in patients with septic shock. The rs1518111 T-allele frequency increased from 0.323 in the non-shock group to 0.785 in the septic shock group, while the rs1554286 A-allele frequency increased from 0.294 to 0.785. Correspondingly, homozygous variant genotypes were substantially more frequent among patients with septic shock, with T/T and A/A each observed in 71.4% of the shock group compared with 14.7% and 11.8%, respectively, in the non-shock group. These findings demonstrate a pronounced genotype and allele-frequency shift between the two clinical phenotypes and are consistent with the strong linkage disequilibrium observed between the two loci (Table 3).

Both variants deviated significantly from HWE, within the non-shock sepsis group (rs1518111, P < 0.001; rs1554286, P = 0.00245). Because this group comprised patients with sepsis rather than an unrelated population-based reference sample, these deviations were not interpreted as evidence of disease association or genotyping failure. The HWE, results were therefore considered in conjunction with the laboratory quality-control procedures, which included blinded genotyping, negative controls, replicate amplification, and reproducibility testing. The marked enrichment of variant homozygotes in the septic shock group may contribute to the observed distributional differences; however, population structure, selection effects, and other sources of deviation cannot be excluded solely on the basis of HWE, testing.

The homozygous variant T-A/T-A diplotype was independently associated with an increased risk of septic shock (aOR, 6.30; 95% CI: 3.68–10.78; *P* < 0.001). Conversely, the homozygous wild-type C-G/C-G diplotype was independently associated with a substantially lower likelihood of septic shock (aOR, 0.28; 95% CI: 0.14–0.61; *P* < 0.001), indicating independent protective association. In contrast, individuals carrying the heterozygous C-G/T-A diplotype exhibited no additional risk beyond the reference category. Collectively, the observed association was concentrated among individuals homozygous for the T-A haplotype, consistent with a diplotype-specific pattern within this cohort; however, the data do not establish a definitive biological inheritance model.

Genotype-phenotype mapping (Table 4) demonstrates a coordinated, dose-dependent relationship between IL-10, variant carriage, cytokine expression kinetics, and hemodynamic stability. Under baseline conditions, homozygous wild-type carriers maintain regulated systemic and localized IL-10, concentrations (median serum ∼165.0 pg/mL) alongside relative hemodynamic stability (mean SBP ∼120.0 mmHg). Heterozygous carriage does not substantially alter this physiological baseline, consistent with a recessive inheritance model.

In contrast, homozygous variant carriage (rs1518111 T/T and rs1554286 A/A) shows a statistically significant hyper-secretory phenotype, yielding peak systemic (median >214.0 pg/mL) and localized urinary (median >19.5 pg/mL) IL-10, concentrations (*P* < 0.001). Crucially, this biochemical surge directly correlates with significant arterial depression. With the scaled statistical power of the cohort, variant homozygotes presented with highly significant hypotensive profiles across the cohort, with SBP, dropping to ∼110.0 mmHg (*P* < 0.001) and DBP, dropping to ∼68.0 mmHg (*P* < 0.01).

These findings are consistent with an association between IL-10, regulatory variation, circulating IL-10, concentrations, and hemodynamic phenotype, but they do not establish a causal pathway between genotype, cytokine production, and circulatory deterioration.

Receiver operating characteristic (ROC) analysis (Table 5) evaluates the clinical utility of combining genetic risk scoring with proximal biochemical markers of systemic counter-regulation. While standard clinical indicators such as WBC, counts (AUC, 0.744) and compartmentalized urinary IL-10 (AUC, 0.736) demonstrate only moderate discriminative capacity, admission-based serum IL-10, showed the highest apparent discrimination among the individual variables evaluated (AUC, 0.851; 95% CI: 0.808–0.894). At an optimal threshold of 210.0 pg/mL, serum IL-10, showed the highest discrimination among the individual variables evaluated (AUC, 0.851; 95% CI, 0.808–0.894), whereas the combined model showed higher apparent discrimination than the individual markers (AUC, 0.889 (95% CI, 0.820–0.958), although these estimates were derived and evaluated in the same cohort and therefore require external validation.

Crucially, integrating this physiological marker with admission WBC, counts and the discrete rs1518111–rs1554286 diplotype risk configurations into an optimized Combined Clinical-Genomic Model significantly improves overall predictive performance. This integrated model achieves a peak AUC, of 0.889, and due to the cohort size (N = 492), the 95% Confidence Interval has narrowed substantially (0.820–0.958), providing a much higher degree of statistical precision. The combined model showed promising discrimination within the present cohort (AUC, 0.889); however, because model development and evaluation were performed in the same population, the observed performance may be optimistic. External validation and preferably prospective recalibration are required before its clinical performance can be established.

## Data Availability

De-identified analytical datasets and statistical outputs are available from the corresponding author upon reasonable academic request.
